# Gluten-Free Diet and Metabolic Syndrome: Could Be a Not Benevolent Encounter?

**DOI:** 10.3390/nu15030627

**Published:** 2023-01-26

**Authors:** Giuseppe Defeudis, Maria Chiara Massari, Giovanni Terrana, Lucia Coppola, Nicola Napoli, Silvia Migliaccio

**Affiliations:** 1 Department of Movement, Human and Health Sciences, University Foro Italico of Rome, 00135 Rome, Italy; 2 Department of Experimental Medicine, Sapienza University of Rome, 00185 Rome, Italy; 3 Unit of Endocrinology and Diabetes, Department of Medicine, University Campus Bio-Medico of Rome, 00128 Rome, Italy

**Keywords:** gluten-free diet, metabolic syndrome, glycemia, inflammation, celiac disease, nutrition

## Abstract

Celiac disease is a rising disorder and is becoming frequently diagnosed in recent years. To date, the only available treatment is the gluten-free diet (GFD). The role of gluten on components of metabolic syndrome and on related inflammatory response is still unclear due to controversial results. In recent years, scientific focus on this topic has been growing up, in particular regarding the role of the GFD on glycometabolic parameters and diabetes. In addition, studies on the remaining components showed discordant results, which was likely due to heterogeneous and large celiac disease populations and to the lack of prospective studies. Furthermore, knowledge about the role of the GFD on inflammatory cytokines and the relationship among vitamin D and celiac disease, metabolic syndrome (MS) and GFD is needed. In this narrative review, we provided evidence regarding the role of the GFD on glycometabolic parameters, cholesterol, triglycerides, waist circumference, blood pressure and inflammatory cascade, also evaluating the role of vitamin D, trying to summarize whether this nutritional pattern may be a value-added for subjects with dysmetabolic conditions. Finally, due to the limited findings and very low-certainty evidence, predominantly based on observational studies, the real effects of a GFD on different components of MS, however, are unclear; nevertheless, an improvement in HDL levels has been reported, although data on glycemic levels are discordant.

## 1. Introduction

Celiac disease (CD) is a disorder, occurring in genetically susceptible subjects, characterized by an abnormal immune reaction to ingested gluten proteins. Its frequency appears to have significantly increased in recent years. To date, the only available treatment in patients affected by CD is the gluten-free diet (GFD). In recent years, scientific interest in the role of the GFD in components of metabolic syndrome (MS) on glycometabolic parameters and diabetes mellitus (DM) has been growing, but results are still unclear due to controversial results in the literature.

In this narrative review, we report evidence regarding the role of the GFD on glycemia, glycometabolic parameters, cholesterol and triglycerides, waist circumference blood pressure and inflammatory parameters, trying to summarize whether this diet may be an added value for subjects with dysmetabolic conditions.

### 1.1. Celiac Disease

CD is increasing in incidence perhaps due to the use of more sophisticated biomarkers and diagnostic tools [1,2]; it occurs only in genetically susceptible subjects in association with environmental factors. Definitive diagnosis of CD is possible when duodenal villous atrophy occurs in the presence of circulating antibodies against tissue transglutaminase.

Among the comorbidities associated with CD is DM and in particular type 1 diabetes (T1D) [3] with prevalence ranging from 3% to 16% in children and from 1.4% to 6.8% in adults [4,5,6,7].

Finally, is noteworthy to mention the important role of the inflammatory cascade linked to this disease, as reported later in the text.

### 1.2. Gluten-Free Diet

The GFD is the currently available treatment for CD and it is and it is unfortunately associated with low intake of dietary fiber, minerals, and vitamins, combined with high intake of calories, carbohydrates, fats, other micronutrients [8] and food with a usually high glycemic index. In particular, the GFD consists of a complete elimination of gluten-containing foods, including gluten proteins in wheat (gliadin), rye (secalins), and barley (hordeins) [9]. In addition, to prevent weight gain, the GFD should be individualized in order to maintain optimal body composition and lead to an eventual reduction in peripheral adipose tissue (when needed), as it is desirable in subjects affected by insulin resistance [10].

According to the Food and Drugs Administration (FDA), a gluten-free product is defined as having <20 ppm (parts per million) of gluten while considering possible contamination during product creation [11,12]. Since 2005, the European Union (EU) has declared that food containing gluten must have listed in the ingredients list, specifying two levels—gluten-free (≤20 ppm/mg/kg) and low gluten (21–100 ppm/mg/kg) [12].

It is important to consider that a GFD inadequately balanced might have serious consequences on the metabolic system and increase the risk of MS [8].

### 1.3. Metabolic Syndrome

MS is a condition characterized by the concomitant presence of three or more metabolic abnormalities, including waist circumference ≥ 94 cm in males or ≥80 cm in females (for WHO and IDF report for Caucasian population), fasting serum glucose >100 mg/dL, triglycerides >150 mg/dL, HDL cholesterol <40 mg/dL in males and <50 mg/dL in females, systolic blood pressure ≥130 mmHg and diastolic blood pressure ≥ 85 mmHg, according to the IDF and AHA/NHLBI representatives; so, the presence of any three of five risk factors constitutes a diagnosis of MS [13,14,15].

## 2. Materials and Methods

This narrative review was performed on all available prospective, retrospective and review studies that have been published since 2000 in PubMed. Data were extracted from the text and tables of the manuscripts. The keywords used in this study were: “gluten-free diet and metabolic syndrome”, “gluten-free diet and blood sugar”, “gluten-free diet and cholesterol”, “gluten-free diet and weight circumference”, “gluten-free diet and blood pressure”, “gluten-free diet and cardiovascular disease”, “celiac disease and metabolic syndrome”, “gluten-free diet and inflammation”, “vitamin D and metabolic syndrome and diseases”, “vitamin D and gluten-free diet and metabolic syndrome”, and “vitamin D and gluten-free diet”.

## 3. Gluten-Free Diet and Components of the Metabolic Syndrome

Due to controversial results and unspecified diet composition in some studies, the role of the GFD on components of MS is still unclear. Therefore, we focused on studies that evaluated the effect of the GFD on all MS components (Table 1) as well as the effect on inflammatory response (Table 2).

### 3.1. Glycemia and Glycemic Parameters

Literature data regarding the effects of the GFD on glycemic parameters are controversial. Studies on subjects with CD and T1D have shown an improvement in fasting blood glucose with a reduction in islet-specific autoantibodies upon administering a GFD [42]. In addition, the relationship among CD and T2D is still not well defined: some studies showed a prevalence comparable with that of healthy subjects, while others showed higher prevalence [8,43,44]. In a prospective open-label randomized 1 year-controlled trial (RCT) on subjects with T1D and subclinical CD treated with a GFD, there was an improvement of glycemic control and a decrease in hypoglycemic episodes compared to subjects in normal diet [16].

In contrast, in 2015, an Italian study showed increased levels of glycemia and altered values of components of MS upon 1 year of evaluation of subjects on a GFD [17,20]. In T1D subjects, asymptomatic but biopsy-confirmed for CD, no HbA1c differences were observed between a GFD and diet with gluten; however, an increase in postprandial glycemia was present in the GFD group [18].

In a recent metanalysis and in a systematic review of the literature, children on a GFD with asymptomatic T1D and CD were evaluated, and no significant effects on HbA1c were observed [19]. Furthermore, in a case-control, observational study, T1D and CD long-term GFD-treated, glycemic control did not worsen [23]. In particular, these studies showed that a GFD has no significant impact on glycemic levels [19,20,21,22]. Moreover, a study performed in 20 hospitals in the Netherlands, designed to evaluate the effect of a GFD on glycemic control in subjects with T1D and CD, demonstrated no changes in terms of reduction in HbA1c [3].

In a large prospective study on children, mostly CD asymptomatic, HbA1c increased by 0.6% after 1 year of GFD treatment [24]; other trials on subjects with CD showed no significant difference in HbA1c [20,25,26,27,28]. In a randomized clinical trial on subjects with MS and CD, treated vs. untreated with a GFD, fasting glycemia decreased significantly in the GFD group [29].

Regarding data on subjects without CD, in an RCT, a GFD appears to improve fasting blood glucose [2]. Interestingly, studies performed in an experimental animal model of T2D mice showed that a GFD improves disease parameters by acting on intestinal barrier function and TLR4 receptor stimulation, where gliadin enhances insulin resistance and β-cell dysfunction [45], thus suggesting a positive role of GFD on T2D mice without CD [46]. In 2017, in a double-blind randomized crossover trial on healthy adults, the postprandial glycemic responses to GFD were compared to wheat pasta. Remarkably, glucose levels were 57% higher compared to traditional wheat pasta [37], suggesting a potential detrimental effect on glucose metabolism. Finally, in 2022, in a systematic review regarding the long-term effect of a gluten-reduced diet or GFD on the prevention of cardiovascular disease (CVD) [36], the authors hypothesized that it is uncertain whether higher gluten intake can increase the risk of developing T2D, as shown in Table 1.

### 3.2. HDL and Triglycerides

To date, the effects of a GFD in CD subjects regarding lipid metabolism and lipid levels are still unclear.

A 2015 study reported that a GFD reduced HDL cholesterol levels [17], whereas in a study on CD children, it showed significantly higher HDL levels in girls than in boys [30], suggesting a gender difference in response to a GFD. A trial in 2016 showed that a GFD induced significantly lower HDL-C levels in children with T1D and CD compared to the control group: indeed, a GFD induced a significant increase in HDL-C (60.9 ± 13.7 vs. 51.3 ± 13.6 mg/dL, *p* < 0.0001) [31]. In contrast, a significant reduction in HDL was found in a retrospective cohort with CD [29].

With regard to triglycerides, Tortora et al., in a prospective case-control study on subjects with CD, found a worsening of triglycerides [17,32], while a retrospective study conducted by Ciccone et al. did not show a significant reduction in triglycerides [29]. Finally, another interesting study performed on children receiving a GFD showed higher triglyceride levels in girls than in boys, indicating that the lipid profiles of children with CD may differ across gender [30,37].

Moreover, in regard to subjects with CD treated with a GFD, Kim et al. [38] and Di Giacomo et al. [47] showed an improvement of HDL levels, whereas another RCT study showed that a GFD in subjects without CD could improve triglycerides levels [2] (Table 1).

### 3.3. Waist Circumference

An important component of MS is an increase in waist circumference (WC). Recently, in a randomized clinical trial, enrolling 50 subjects with MS and CD, and randomly divided into a group receiving a GFD and a group continuing a regular diet, the normocaloric GFD reduced WC compared with the control diet group (*p* < 0.05) [2]. In contrast, some studies revealed that a GFD worsened WC [17]. In particular, in a population with CD, WC increased upon GFD nutritional intervention [17].

In subjects without CD on a GFD, a study showed an improvement in WC [38]. Interestingly, an improvement in WC was also demonstrated by data obtained from the National Health and Nutrition Examination Survey (NHANES) 2009–2014 in non-CD subjects [33,38] (Table 1).

### 3.4. Blood Pressure

Blood pressure is an MS component that needs to be evaluated, and it may depend on an unbalanced dietary pattern. Interestingly, high blood pressure was observed in subjects with CD after 1 year of GFD [17]. As mentioned above, in a study performed on children with CD and receiving a GFD or newly diagnosed children, the first group had blood pressure significantly higher in girls than in boys also compared with the group without a GFD [30]. Furthermore, in a prospective study conducted to assess the effects of a GFD on CVD risk among newly diagnosed pediatric CD subjects, no significant data were displayed in regard to changes in blood pressure [34]. In a randomized clinical trial, evaluating subjects with MS and CD, a group treated with GFD and the other with regular diet, no significant differences were reported regarding systolic and diastolic blood pressure values [29]. A systematic review of the literature showed no modifications of blood pressure after adhering to a GFD in CD subjects [35]. Furthermore, in a case-control observational study performed on 34 T1D CD patients in comparison to 66 patients with T1D alone, both groups GFD-treated, no differences were found in systolic blood pressure, while diastolic blood pressure was significantly lower in T1D CD subjects (*p* = 0.003) [23].

A recent revision of the literature, evaluating in subjects without CD the effects of a gluten-reduced diet or GFD on primary prevention of CVD, showed not significant differences in either systolic or diastolic blood pressure after six months of follow-up [36], as shown in Table 1.

## 4. Gluten-Free Diet and Inflammation

The presence of MS leads to a chronic low-grade inflammation and to an activation of the immune system, which is a potential significant pathogenetic factor of obesity-related insulin resistance and T2D. Indeed, an increase in macrophages and other immune cells in involved in a pro-inflammatory action via cytokines alterations in main tissues and organs [48].

As known, CD is related to an immune response driven by CD4+ T cells specific for deamidated gluten peptides; in fact, these molecules bind to disease-associated human leukocyte antigen (HLA)–DQ allotypes, where the CD4 T cells interact with B cells and also CD8 T cells [49,50]. This increase in T cell subtypes activates specific signaling including NF-kB, GATA1, JAK or STAT5, which is targeted by diet or drugs. Furthermore, IL-15 also plays a central and targeted role in this process by activating CD8+ T cells via CD4+ T cells [51].

Additionally, other possible pathological pathways could be involved, such as the activation of the innate immune response via the α-gliadin peptide, p31-43 peptides, directly damaging the CD mucosa, which is supported by the activation of neutrophils, eosinophils, complement proteins and mast cells [52,53,54].

It is interesting that an important role in balancing the cascade of inflammation is performed by prebiotics and probiotics, stimulating the metabolic activity of gut microbiota with beneficial effects on the immune response and cytokine production [55,56,57]. Indeed, among probiotics, the supplementation of Bifidobacterium and Lactobacilli showed a decrease in inflammation via a reduction in cytokine and antibody production. Nonetheless, the use of prebiotics and probiotics in subjects with CD is not yet formally allowed in clinical practice since no clinical RCT have been performed yet [58].

Studies on subjects with irritable bowel syndrome (IBS) have indicated that gluten exposure leads to monocyte and cytokine production and intestinal low-grade inflammation, whereas no data on gluten-induced inflammation have been produced as yet [41], as shown in Table 2.

In NCG/WS (non-celiac gluten or wheat sensitivity), a GFD restores the microbiota population and reduces pro-inflammatory species. Innate and adaptive immunity coexist in these conditions: the first one shows an increase in mucosal toll-like receptor 2 (TLR2), granulocyte colony-stimulating factor (GCSF), IL-10, transforming growth factor alpha (TNF-α) and CXCL-10 chemokine from peripheral blood mononuclear cells (PBMCs), while the second one increases interferon (IFN)-gamma mRNA [39]. Finally, in a study using a GFD and dairy-free diet in children with steroid-resistant nephrotic syndrome (SRNS), findings showed a decrease in the inflammatory status of this population [40].

## 5. The Role of Vitamin D in Metabolic Syndrome, Celiac Disease and in Gluten Free Diet Treatment

Vitamin D is a hormone which plays a pivotal action on calcium and bone metabolism. In the adult population, serum levels of vitamin D in its hydroxylated form (25-hydroxy-vitamin D or calcidiol) are defined as deficient when they are below 20 ng/mL and insufficient when they are between 20 and 30 ng/mL [59]. Since low levels of vitamin D are not uncommon in the general population and its supplementation is becoming increasingly popular, great attention has been paid to this hormone and its receptor, as well as a condition of hypovitaminosis D (HypoD), in regard to extra-skeletal effects as well as being a co-factor in several diseases. In particular, several studies have investigated the relationship between vitamin deficiency and components of MS.

### 5.1. Vitamin D and MS

Vitamin D deficiency appears to be bidirectional related to obesity: on the one hand, the excess adipose mass might act as a storage site for a lipophilic hormone such as vitamin D [60]; on the other hand, vitamin D plays an important role in adipocyte physiology and glucose metabolism, which is typically dysregulated in obese subjects [61]. Although there has been some heterogeneity in the literature, most studies confirm an inverse correlation between vitamin D levels and the prevalence of MS. HypoD has been associated with increased risk or prevalence of abdominal obesity, hypertension, impaired glucose homeostasis, dyslipidemia, and increased waist circumference [62,63,64,65,66,67,68,69]. Recently, a cross-sectional study on more than 800 participants, aged > 60, showed a reduced risk of MS above specific serum vitamin D cut-off levels, which was different according to sex, 40 ng/mL in men and 20 mg/dl in women, respectively [70]. Additionally, a large survey of nearly 10,000 participants identified HypoD as an additional risk factor to the presence of MS for the development of cardiovascular events and all-cause mortality, which were shown to be inversely related to vitamin D levels [71]. Finally, it appears noteworthy to evaluate the relationship between vitamin D and CD, the prevalence of HypoD among subjects with CD compared to subjects without CD, and the effect of a GFD on levels of the hormone, which appears to play an important role in the development and progression of MS.

### 5.2. Vitamin D and CD

As previously mentioned, CD is a chronic auto-immune and self-inflammatory condition that damages intestinal tissues with alterations in intestinal villi and crypts and indirectly with altered absorption of various nutrients, leading to malnutrition [72]. Micronutrient deficits are common in individuals with CD, during both childhood and adulthood, although data in the literature are heterogeneous [73]. Notably, among micronutrients, several guidelines recommend the measurement of vitamin D levels at the time of CD diagnosis [74,75]. The link between CD and HypoD is not entirely clear: since vitamin D has an immunomodulatory and controlling action on intestinal permeability, it has been hypothesized that low levels of this hormone may play a role in the pathogenesis and development of CD by counteracting the mechanisms leading to the onset of intestinal autoimmunity [76]. The prevalence of HypoD in patients with CD varies among studies: low levels appear to be significantly related to CD in pediatric populations [77,78], while differences are smaller as compared to healthy controls when considering adult patients [73]. However, CD, as other inflammatory bowel diseases, is also linked to alterations in bone metabolism, so celiac subjects have lower BMD values than non-celiac subjects (up to 70% of cases in some studies) [79].

### 5.3. Vitamin D and GFD

The effect of a GFD on vitamin D levels in CD subjects has been evaluated in several studies. In regard to pediatric case, it appears that celiac children, observing a GFD, have dietary trends similar to the corresponding healthy controls, with both celiac and non-celiac subjects having vitamin D intake below reference standards [80,81]. However, in two prospective studies on pediatric celiac patients, after one year or six months of GFD, respectively, a significant increase in vitamin D level was observed, which was associated or not with a decline in PTH levels [82,83]. Similar results were obtained in a cohort of the adult population, where patients not adhering to a GFD had lower vitamin D and BMD values at diagnosis, and higher PTH values than subjects adhering to the diet [84]. While lower levels of vitamin D are more common in newly diagnosed CD or untreated subjects, it has been seen that HypoD may also be found in those on a GFD [85,86]. The reason could be due to nutritional deficits caused by malabsorption in celiac patients with bowel damage [87]. According to recent evidence, the deficit would persist regardless of the duration and adherence to the GFD, being mainly related to poor intake of the nutrient itself, which in 95% of cases is not added to gluten-free products [88]. Despite this, it has been suggested that vitamin D supplementation is essential in those subjects undergoing dietary treatment, especially during the first year of the GFD [89].

## 6. Discussion

The evaluation of the effects of a GFD on each component of the MS is a measure to consider in the prevention of potential increasing risk for cardiovascular diseases in general and, in particular, in specific populations, such as those with DM, overweight or obesity, even evaluating the impact on inflammation. To date, several trials have evaluated the role of a GFD on components of MS, mainly in CD, less in those without CD. and to the best of our knowledge, there are controversial results, with few RCT and intervention trials of longer duration and larger sample size.

### 6.1. Summarizing Controversial Data on Components of MS

**Figure 1:**
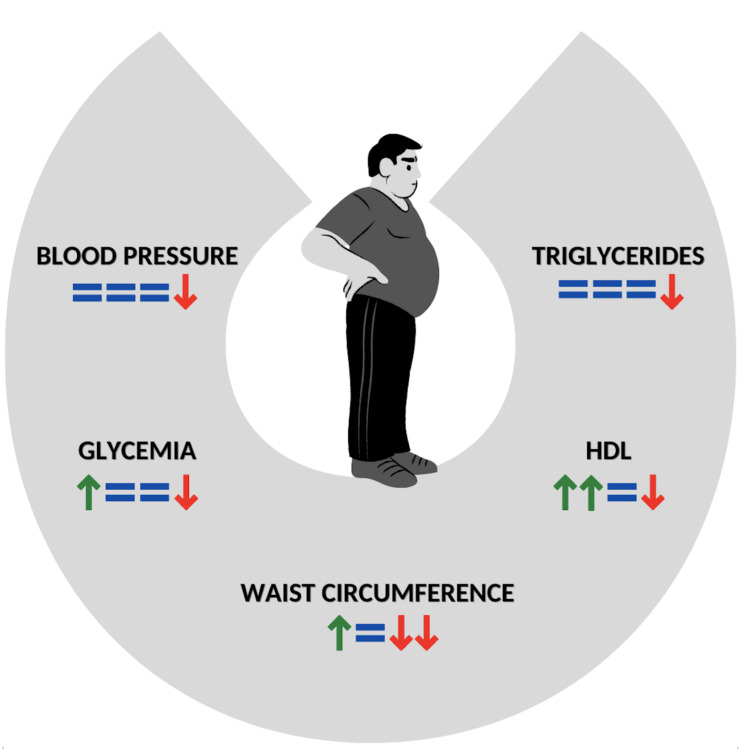
Impact of GFD on components of MS in subjects with CD. Green arrow: improvement of component; Red arrow: worsening of component; =: not significant data. The number of arrows means in a scale 1–4 the power of data. GFT: Gluten-free diet; MS: metabolic syndrome; CD: celiac disease; HDL: HDL Cholesterol.

To date, these controversial and inconclusive data might also be derived from a poor detailed description of GFD composition, which could be considered a bias when results of different studies are compared. Summarizing this discrepancy in a few reports: Tortora et al., treating subjects affected by CD with a GFD, showed an elevated risk of MS after one year [17]; whereas De Marchi et al., in a study on young adults with CD, demonstrated that a GFD improved HDL, IMT and endothelium-dependent dilatation [90]. In non-celiac patients, a GFD may slightly improve overall cardiac risk factors; nevertheless, its use in the absence of a gluten-related disease needs larger studies with a better-defined dietary composition of food [8] (Figure 1).

### 6.2. Potential Risks of GFD Using for Non-Celiac Subjects

To date, a GFD has not proved to be beneficial in subjects without CD [8]; therefore, we should not consider a GFD in non-CD patients to promote weight loss or improve triglycerides. Finally, considering the scientific evidence, although discordant, it is fair to conclude that subjects without CD or gluten intolerance, might not choose a GFD in place of traditional foods to obtain metabolic improvement or prevention of MS.

### 6.3. Future Perspectives

Even if in recent years, the use of the GFD is growing, not only in subjects with CD, the components of MS still remain one of the main topics to focus to improve the reduction in cardiovascular risks. Anyhow, there is a gap of knowledge to fill. In this direction, it could be useful to organize further evaluations with larger RCT of longer duration with a large sample size, possibly including evaluation on glycemic variability, using continuous glucose monitoring systems (CGMs) to better evaluate subjects with prediabetes or diabetes to optimize treatment. Furthermore, a clear and well-characterized dietary composition in all studies is needed. Finally, it could be useful to promote a more complete evaluation of the effects of the GFD on inflammatory pathways, bone metabolism and fractures, microbiome imbalance and sexual dysfunctions.

Hence, the proposed path is to achieve a GFD tailored to the type of subjects with DM, CVD, bone disease, etc.

## 7. Conclusions

Given the limited findings in the literature on the GFD, predominantly based on observational studies, few definitive conclusions can be drawn on the real effects of this nutritional pattern on different components of MS. With this narrative review of the literature, we report that data on blood glucose and waist circumference are discordant; HDL levels seem to be improved while triglyceride levels are modulated by the use of the GFD. Finally, blood pressure is increased by the GFD in most of the studies evaluated, and vitamin D supplementation should be assessed in CD subjects on the GFD. In conclusion, there are still multiple aspects to explore and to characterize, and further studies are needed to better define the effects of a GFD in subjects without CD, such as a potential role of microbiome axis as well cardiovascular risk factors, diabetes complications and osteometabolic diseases.

## Figures and Tables

**Table 1:** Summary of effects of GFD on components of metabolic syndrome in studies on subjects with and without celiac disease.

Study Name	Study Design	CD/no CD	Cases	Characteristics of GFD	Glycemia	Triglycerides	HDL	Waist Circumference	Blood Pressure
Kaur et al., 2020 [16]	Open label randomized controlled trial.	CD	30	Patients were given a list of safe and unsafe food products	Improved glycemic control	Not available	Not available	Not available	Not available
Tortora et al., 2015 [17]	Prospective cohort study	CD	98	Removal of gluten Gluten-free products used	Increased	Increased	No significant changes	Increased	Increased
Mahmud et al., 2020 [18]	Randomized clinical trial.	CD	51	Gluten-free diet not otherwise specified	Greater postprandial glucose excursions	Not available	Not available	Not available	Not available
Burayzat et al., 2022 [19]	Meta-analysis and systematic review	CD	578	Gluten-free diet, not otherwise specified	No significant positive or negative effect	No significant positive or negative effect	No significant positive or negative effect	Not available	Not available
Hansen et al., 2006 [20]	Clinical trial (population based study)	CD	31	Gluten-free diet, not otherwise specified	No significant changes	Not available	Not available	Not available	Not available
Wherrett et al., 2013 [21]	Clinical Practice Guidelines	CD	-	Gluten-free diet, not otherwise specified	No significant positive or negative effect	No significant positive or negative effect	No significant positive or negative effect	No significant positive or negative effect	No significant positive or negative effect
DeMelo et al., 2015 [22]	Review	CD	-	Gluten-free diet, not otherwise specified	No significant positive or negative effect	Not available	An increase in HDL was noted	May be beneficial	Not available
Creanza et al., 2018 [23]	Case-control study	CD	100	Removal of gluten Gluten-free products used	No significant changes	No significant changes	No significant changes	Not available	Not available
Bakker et al., 2013 [3]	Clinical trial	CD	77	Removal of gluten	No significant changes in HbA1c	No significant changes	No significant changes	Not available	No significant changes
Sun et al., 2009 [24]	Clinical trial	CD	98	Removal of gluten	Increase in HbA1c	Not available	Not available	Not available	Not available
Saadah et al., 2004 [25]	Clinical trial	CD	63	Removal of gluten Gluten-free products used	No significant difference in HbA1c	Not available	Not available	Not available	Not available
Abid et al., 2011 [26]	Open label clinical trial	CD	22	Gluten-free diet, not otherwise specified	No significant difference in HbA1c	Not available	Not available	Not available	Not available
Sanchez-Albisua et al., 2005 [27]	Open label clinical trial	CD	9	Gluten-free diet, not otherwise specified	Improved glycemic control	Not available	Not available	Not available	Not available
Taler et al., 2012 [28]	Longitudinal observational case–control study	CD	199	Removal of gluten Gluten-free products used	No significant difference in HbA1c	Not available	Not available	Not available	Not available
Ciccone et al., 2019 [29]	Open label clinical trial	CD	185	Removal of gluten Gluten-free products used	Increased	No significant changes	Reduced	Increased	Increased
Forchielli et al., 2015 [30]	Cross-sectional study	CD	235	Removal of gluten, reduced use of gluten-free products	Not available	Not available	Not available	Not available	Not available
Salardi et al., 2016 [31]	Review	CD	-	Gluten-free diet, not otherwise specified	Not available	Not available	HDL-C increased and normalized	Not available	Not available
Tortora et al., 2018 [32]	Case-control study	CD	377	Removal of gluten Gluten-free products used	Not available	Not available	Not available	Not available	Not available
Emilsson et al., 2017 [33]	Editorial on a cross-sectional study	CD	-	-	No significant difference	No significant difference	Higher in GFD followers	Smaller in GFD followers	No significant difference
Zifman et al., 2019 [34]	Open label clinical trial	CD	110	Gluten-free diet, not otherwise specified	Increased fasting glycemia	No significant changes	Increased	No significant changes	No significant changes
Potter et al., 2018 [35]	Systematic review	CD	872	Gluten-free diet, not otherwise specified	Increased fasting glycemia	No significant difference	Increased	Increased	No significant difference
Dhruva et al., 2021 [8]	Review	With and without CD	-	Gluten-free diet, not otherwise specified	Improved glycemic control	Unclear	Unclear	Not available	Unclear
Schmucker et al., 2022 [36]	Review	With and without CD	-	Gluten-free diet, not otherwise specified	Not available	Not available	Not available	Not available	Unclear
Johnston et al., 2017 [37]	Randomized crossover trial	no CD	13	Rice and corn flour pasta	Higher postprandial glycemia	Not available	Not available	Not available	Not available
Ehteshami et al., 2018 [2]	Randomized clinical trial.	no CD	45	Removal of gluten 1250 g of gluten-free bread per week	Improved glycemic control	No significant changes	No significant changes	Not available	No significant changes
Kim et al., 2017 [38]	Cross-sectional study	no CD	-	Gluten-free diet, not otherwise specified	No significant difference	No significant difference	Higher in GFD followers	Smaller in GFD followers	No significant difference

CD: celiac disease; GFD: Gluten-free diet; HDL: HDL Cholesterol; HbA1c: Glycated hemoglobin.

**Table 2:** Studies about the effects of inflammation on subjects treated with GFD.

Study Name	Study Design	Cases	Characteristics of GFD	Effect on Inflammation
Caio et al., 2020 [39]	Review	-	Gluten-free diet, not otherwise specified	Reduced
Pérez-Sáez et al., 2021 [40]	Prospective intervention study	16	Gluten-free diet, dairy free diet, low sodium (<800 mg/day)	Reduced
Palmieri et al., 2019 [41]	Review		Gluten-free diet, not otherwise specified	Reduced

## Data Availability

Not applicable.
